# Co-expression of TIMP-1 and its cell surface binding partner CD63 in glioblastomas

**DOI:** 10.1186/s12885-018-4179-y

**Published:** 2018-03-09

**Authors:** Charlotte Aaberg-Jessen, Mia D. Sørensen, Ana L. S. A. Matos, José M. Moreira, Nils Brünner, Arnon Knudsen, Bjarne W. Kristensen

**Affiliations:** 1 Department of Pathology, Odense University Hospital, J.B. Winsloews Vej 15, 5000 Odense, Denmark; 2 Department of Nuclear Medicine, Odense University Hospital, Sdr. Boulevard 29, 5000 Odense, Denmark; 3 Department of Clinical Research, University of Southern Denmark, J.B. Winsloews Vej 19, 5000 Odense, Denmark; 4 Cancer Research Group, Department of Drug Design and Pharmacology, Faculty of Health and Medical Sciences, University of Copenhagen, Strandboulevarden 49, 2100 Copenhagen, Denmark

**Keywords:** TIMP, CD63, LAMP-3, Glioblastoma, Glioma, Astrocytoma, Microglia, Extracellular matrix, Prognosis, Biomarker

## Abstract

**Background:**

We have previously identified tissue inhibitor of metalloproteinases-1 (TIMP-1) as a prognostic marker in glioblastomas. TIMP-1 has been associated with chemotherapy resistance, and CD63, a known TIMP-1-binding protein, has been suggested to be responsible for this effect. The aim of this study was to assess CD63 expression in astrocytomas focusing on the prognostic potential of CD63 alone and in combination with TIMP-1.

**Methods:**

CD63 expression was investigated immunohistochemically in a cohort of 111 astrocytomas and correlated to tumor grade and overall survival by semi-quantitative scoring. CD63 expression in tumor-associated microglia/macrophages was examined by double-immunofluorescence with ionized calcium-binding adapter molecule 1 (Iba1). The association between CD63 and TIMP-1 was investigated using previously obtained TIMP-1 data from our astrocytoma cohort. Cellular co-expression of TIMP-1 and CD63 as well as TIMP-1 and the tumor stem cell-related markers CD133 and Sox2 was investigated with immunofluorescence. TIMP-1 and CD63 protein interaction was detected by an oligonucleotide-based proximity ligation assay and verified using co-immunoprecipitation.

**Results:**

The expression of CD63 was widely distributed in astrocytomas with a significantly increased level in glioblastomas. CD63 levels did not significantly correlate with patient survival at a protein level, and CD63 did not augment the prognostic significance of TIMP-1. Up to 38% of the CD63+ cells expressed Iba1; however, Iba1 did not appear to impact the prognostic value of CD63. A significant correlation was found between TIMP-1 and CD63, and the TIMP-1 and CD63 proteins were co-expressed at the cellular level and located in close molecular proximity, suggesting that TIMP-1 and CD63 could be co-players in glioblastomas. Some TIMP-1+ cells expressed CD133 and Sox2.

**Conclusion:**

The present study suggests that CD63 is highly expressed in glioblastomas and that TIMP-1 and CD63 interact. CD63 does not add to the prognostic value of TIMP-1. Co-expression of TIMP-1 and stem cell markers as well as the wide expression of CD63 might suggest a role for TIMP-1 and CD63 in glioblastoma stemness.

**Electronic supplementary material:**

The online version of this article (10.1186/s12885-018-4179-y) contains supplementary material, which is available to authorized users.

## Background

Glioblastomas are the most common primary brain tumors in adults and among the most lethal cancers. Despite aggressive treatment consisting of surgery, radiation, and temozolomide, glioblastomas remain incurable [1]. Complete surgical resection is almost impossible to achieve, and it is well-known that glioblastomas are partly resistant towards therapy possibly explaining the dismal patient prognosis. Especially, the immature tumor-initiating cells – the supposed tumor stem-like cells – may explain the apparent resistance towards radio- and chemotherapy [2–5]. The mechanisms behind this resistance are complex with multiple contributing factors. One of these factors could be tissue inhibitor of metalloproteinase 1 (TIMP-1) as TIMP-1 is involved in different cellular survival functions [6–8] and has been associated with decreased chemo-sensitivity in breast and colorectal cancer [9–13].

Using immunohistochemistry we have previously shown that TIMP-1 levels increase with World Health Organisation (WHO) malignancy grade in patients with astrocytoma, and that low levels of TIMP-1 predict a significantly longer overall survival compared with moderate or high TIMP-1 levels in patients with glioblastoma [14]. Similarly, high TIMP-1 levels were reported to correlate with poor prognosis in colorectal [15–18], breast [19–23], gastric [24, 25], and ovarian cancer [26, 27].

Jung et al. showed that TIMP-1 interacts with the cell surface protein CD63, also known as Lysosomal Associated Membrane Protein 3 (LAMP-3), and integrin β1 on the surface of the breast cell line MCF10, which prevented chemo-induced apoptosis [28] suggesting that interaction between TIMP-1 and CD63 may contribute to chemo-resistance. CD63 is a tetraspanin that interacts with various proteins such as integrins and tyrosine kinases involved in regulation of intracellular signal transduction pathways controlling cell adhesion, motility, and survival [28–31]. The CD63 protein is also enriched in exosomes [32]. Reportedly, exosomes contribute significantly to the intercellular communication also in gliomas [32–35] and may play a role in chemo-resistance in cancer [35, 36]. Only few studies have investigated the presence of CD63 in gliomas [37, 38]. Using tissue microarrays, Rorive et al. found the highest level of CD63 expression in pilocytic astrocytomas followed by glioblastomas and anaplastic astrocytomas with the lowest expression in diffuse astrocytomas [37]. Further, CD63 was found to interact with TIMP-4, and CD63/TIMP-4 co-expression was associated with poorer outcome in glioblastoma patients. In contrast, Kase et al. found high amounts of CD63+ immune cells was correlated with better survival following postoperative radiotherapy in patients with glioblastoma [38].

Whether TIMP-1 and CD63 can interact on the surface of glioblastoma cells is so far unknown. A study by Lee et al. showed that glioma-derived TIMP-1 can promote migration of neural stem cells towards the tumor by interacting with CD63 expressed by the stem cells. Similar results were reported for CD34+ hematopoietic stem and progenitor cells (HSPCs), where CD63 together with integrin β1was identified as a receptor complex for TIMP-1 mediating cellular adhesion and migration [39] as well as clonogenic expansion and survival in HSPCs [40]. Collectively, these results suggest a close co-operation between TIMP-1 and CD63 [41].

The aim of this study was to assess the prognostic impact of CD63 alone and in combination with TIMP-1 by performing immunohistochemistry on a cohort of 111 astrocytomas,  described and used previously for evaluation of the prognostic potential of TIMP-1 [14]. Co-expression patterns of TIMP-1 and CD63 were investigated using double-immunofluorescence, proximity ligation assay, and co- immunoprecipitation. The possible co-expression of CD63 and the microglial/macrophage marker ionized calcium-binding adapter-molecule 1 (Iba1) was examined using double-immunofluorescence. To investigate the association between TIMP-1 and cancer stemness, double-immunofluorescence stainings were performed using sex determining region Y-box 2 (Sox2) [42, 43] and CD133 [5, 44–47] as markers of tumor stem-like cells.

## Methods

### Patient tissue

The immunohistochemical expression of CD63 was assessed in archive tumor material obtained from 111 patients who underwent initial surgery for primary glioma between 1995 and 2005 at Odense University Hospital, Denmark. None of the patients had received treatment prior to craniotomy. The patient cohort has been described in previous studies [14, 44, 48, 49]. All tumors were re-classified according the 2016 World Health Classification (WHO) [1]. The cohort included 23 diffuse astrocytomas, 14 anaplastic astrocytomas, and 74 glioblastomas. Patient characteristics are described in Table 1.

**Table 1:** Patient characteristics

	Diffuse astrocytoma	Anaplastic astrocytoma	Glioblastoma
Patients (n)	23	14	74
Age			
Mean	45.0	52.2	61.4
Range	2.6–78.5	29.9–77.3	21.2–78.4
Sex (n)			
Male	15	8	48
Female	8	6	26
Status (n)			
Alive	4	0	1
Dead	19	14	73
Over survival (months)			
Median	57.1	18.4	8.4
Range	3.4–267.0	2.1–48.3	0.08–163.5
IDH status^a^			
Mutated	14	4	2
Wildtype	9	10	72

^a^IDH status was only investigated by immunohistochemistry for glioblastoma patients. For patients diagnosed with diffuse and anaplastic astrocytoma, IDH status was investigated by immunohistochemistry and next generation sequencing (See Materials and Methods section: *Immunohistochemistry*)

*Abbreviations*: *IDH* isocitrate dehydrogenase

### In vitro culturing of spheroids

In vitro culturing of five different patient-derived spheroids was performed using fresh tumor tissue from five patients who underwent initial surgery for astrocytoma WHO grade III and IV from September 2007 through May 2008 at the Department of Neurosurgery, Odense University Hospital, Denmark. Written informed consent was obtained from all five patients. The tumor tissue was processed and cultured as described earlier [50]. In brief, tumor tissue was sectioned manually into small fragments of 200–400 μm using two scalpels. The tumor fragments were cultured for 10–15 days in 0.75% agar-coated culture flasks of 75 cm^2^ containing 20 mL Dulbeccos Modified Eagle Medium (Sigma Aldrich, USA) supplemented with 10% fetal calf serum (Fisher Scientific, USA), 2% glutamine (Cambrex, USA), 4% nonessential amino acids (Cambrex), and 2% penicillin/streptomycin (Cambrex) until spheroids were formed in a standard tissue culture incubator (95% humidity, 95% air, and 5% CO_2_). Commercial U87MG cell line-derived spheroids were cultured under the same conditions until reaching a size of 200–400 μm. Spheroids were then fixed in 4% neutral buffered formaldehyde for 24 h and embedded in paraffin. Afterwards, the preservation and expression patterns of TIMP-1, CD63, and the stem-related markers CD133 and Sox2 were investigated by immunofluorescence and confocal microscopy.

### Immunohistochemistry

Formaldehyde-fixed, paraffin-embedded tissue was cut into three μm sections on a microtome and stained routinely with haematoxylin-eosin to define representative tumor regions. For immunohistochemical staining with CD63, paraffin sections were then deparaffinized, and endogenous peroxidase activity was quenched followed by heat-induced epitope retrieval (HIER) in TRS buffer (Dako, Denmark). The sections were subsequently incubated for 60 min with a CD63 monoclonal antibody (MX-49.129.5, Santa Cruz, 1 + 1000, USA). The antigen-antibody complex was detected using anti-mouse EnVision (Dako) and visualised with diaminobenzidine (DAB) as chromogen. Finally, the sections were counterstained with Mayer’s haematoxylin.

Immunohistochemical staining for mutation in isocitrate dehydrogenase 1 (IDH-1) was performed as previously described [51, 52]. For diffuse and anaplastic astrocytomas that were IDH-1 wildtype by immunohistochemistry, next generation sequencing was performed using the 20-gene panel as reported by Zacher et al. [53].

### Scoring of the CD63 staining

The immunohistochemical scores were based on the average percentage of CD63+ tumor cells, CD63+ tumor blood vessels as well as the overall staining intensity for both tumor cells and blood vessels (Table 2). Necrotic areas as well as invasion zones were excluded. To obtain a total CD63 score for each patient, the scores for staining positivity and intensity of both tumor cells and blood vessels were summed resulting in a maximum total CD63 score of 12. For comparison with overall patient survival, the tumors were divided into three groups corresponding to a low (total CD63 scores 0–6), medium (total CD63 scores 7–8), and high (total CD63 scores 9–12) CD63 expression. The scoring system and the scoring process were developed and was carried out under the supervision of an experienced neuropathologist.

**Table 2:** Assessment of CD63 immunohistochemical staining

Score	0	1	2	3
Positive tumor cells	0% to < 2%	2% to < 30%	30% to < 65%	65% to 100%
Tumor cell intensity	None	Faint	Moderate	Intense
Positive blood vessels	0% to < 2%	2% to < 30%	30% to < 65%	65% to 100%
Blood vessel intensity	None	Faint	Moderate	Intense

All scores were summed into a total CD63 score with maximum score of 12. Based on the total CD63 score, patients were grouped into three categories: 0–6 (low), 7–8 (medium), and 9–12 (high)

### Immunofluorescence and automated quantitative image analysis

To investigate the expression of CD63 in immune cells, double-immunofluorescence was performed on archive tumor material from ten glioblastoma patients. The preparations and the first steps in the stainings are as described above. Following detection of CD63 (NK1/C3, 1 + 50, Abcam, United Kingdom) using Catalyzed Signal Amplification (CSA) II kit with fluorescein (Dako), sections were incubated with an antibody against Iba1 (019–19,741, 1 + 12,000, Wako Pure Chemical Industries, Japan) followed by detection with Tyramide Amplification Signal (TSA)-Cyanine-5 (Perkin Elmer, USA). Nuclei were counterstained with 4.6-diamidino-2-phenylindole (DAPI) (VWR International, USA). Image acquisition and analysis were performed using a Visiopharm integrated Leica DM6000B microscope and software module (Visiopharm, Denmark) connected to an Olympus DP72 1.4 Mega Pixel CCD camera (Olympus, Japan) using DAPI (Omega XF06, Omega Optical, USA), fluorescein (Leica, Germany), and Cyanine-5 (Omega XF110–2) filters. Overview images were acquired using bright field settings. Sampling regions containing vital tumor tissue was manually outlined, and sample images were taken with a 20× objective. Images were quantified using a threshold-based algorithm developed in the Visiopharm software defining the total nuclear area using the DAPI signal. Nuclei surrounded by the Iba1 and/or the CD63 signal were then identified as positive or double-positive cells. Area fractions were then measured calculating the area of double-positive cells as fractions of the total Iba1+ cell area or of the total CD63+ cell area.

### Immunofluorescence and confocal microscopy

HIER was performed in TEG buffer (10 mmol/L Trisbase and 0.5 mmol/L EGTA) followed by incubation for 60 min with a TIMP-1 (VT7 [54], 1 + 4000) or a CD133 (W6B3C1, 1 + 40, Miltenyi, Belgium) antibody. The primary antibody was detected using the CSA II kit (Dako) (TIMP-1 and CD133) or TSA Plus System (Perkin Elmer) (TIMP-1). HIER was repeated followed by incubation for 60 min with an antibody against Sox2 (245,610, 1 + 800, R&D Systems, USA), CD63 (MX-49.129.5, 1 + 800, Santa Cruz), or TIMP-1 (VT7, 1 + 4000). A secondary antibody with Alexa488 (Invitrogen, USA) was used for Sox2, whereas TSA (Perkin Elmer) was used for TIMP-1 and CD63. Nuclei were counterstained with DAPI. The double-immunofluorescence stainings were analysed using a Nikon Eclipse TE2000-E inverted confocal microscope.

### Proximity ligation assay

Detection of TIMP-1 and CD63 protein interaction was investigated using Duolink In Situ (OLINK Bioscience, Sweden) according to manufacturer’s instructions. Paraffin sections were deparaffinized, and endogenous peroxidise activity was quenched followed by HIER in 10 mM citrate buffer (pH 6). Next, sections were incubated with a monoclonal mouse TIMP-1 antibody (VT7 clone [54]) and a polyclonal rabbit CD63 antibody (HPA010088, Atlas Antibodies, Sweden) for 60 min. Secondary antibodies conjugated with oligonucleotides (proximity ligation assay probes) were added, and the sections were incubated for another 60 min at 37 °C. A solution containing ligase oligonucleotides was added for 15 min at 37 °C to bind and ligate the probes together in a closed circle. Ligation would only occur if the probes were located in close proximity. Finally, amplification solution was added for 90 min at 37 °C to amplify the closed ligated circle, leaving a distinct fluorescent spot. Coverslips were mounted using DAPI to visualize cell nuclei. Omission of primary antibodies was used as negative control.

### Immunoprecipitation

For CD63 immunoprecipitation, U87MG cell line-derived spheroids were cultured as described above until reaching a size of 200–400 μm. Cells were washed twice with ice-cold PBS and lysed with lysis buffer (50 mM HEPES, pH 7.4, 150 mM NaCl, 10% glycerol, 1% Triton X-100, 1 mM EDTA) containing phosphatase and protease inhibitor mixtures (Roche Applied Science, Germany). Lysates were precleared for 2 h with Protein G-Dynabeads (Invitrogen) and then incubated overnight with CD63 antibody (clone MX-49.129.5, Santa Cruz) or isotype control prebound to Protein G-Dynabeads. The resin was then washed three times with ice-cold lysis buffer, resuspended in 30 μl of Laemmli sample buffer, boiled for 3 min and centrifuged at 14,000 g for 5 min. The supernatants were analysed by immunoblotting under standard conditions.

### The cancer genome atlas (TCGA) research network

The messenger RNA (mRNA) expression levels of CD63, Iba1 (also known as allograft inflammatory factor 1 (AIF1)), and TIMP-1 were examined in astrocytomas using GlioVis (http://gliovis.bioinfo.cnio.es) [55, 56]. Data were accessible for 327 patients for analysis of the correlation between CD63 and malignancy grade [55]. For survival analyses, data were available for 497 glioblastoma patients [56]. Survival analyses were performed using the median or the 25th percentile as cutoff values.

Differential expression analysis was done using the TCGA to visualize quantitative changes in mRNA expression levels of 12,700 genes between the group of glioblastomas with the highest mRNA level of CD63 (*n* = 123) and the group of glioblastomas with the lowest level (*n* = 125). Gene Ontology (GO) term enrichment analysis was performed for the differentially upregulated and differentially downregulated genes using the AmiGO 2 Term Enrichment Service (http://amigo.geneontology.org/amigo, accessed September 30, 2017) powered by PANTHER (GO Ontology database, Released 2017–09-26) [57–59].

### Statistical analysis

ANOVA with Bonferroni correction was used for comparison of CD63 scores with tumor grade. Student’s unpaired t-test was used to compare differences in CD63 levels in IDH wildtype and IDH mutated tumors. Overall survival was defined from the day of initial surgery until death or date of censoring (1 August 2017). Survival data were analyzed using Kaplan Meier and the multivariate Cox regression model to adjust for age, sex, and TIMP-1 scores. Survival curves were compared using the log-rank test. Spearman’s correlation was used to investigate the association between CD63 and Iba1 and between CD63 and TIMP-1. The statistical analyses were performed in STATA (StataCorp LP, USA) or GraphPad Prism 5.0 (GraphPad Software Inc., USA). *P* < 0.05 was considered significant.

## Results

### CD63 expression in diffuse astrocytomas

**Fig. 1:**
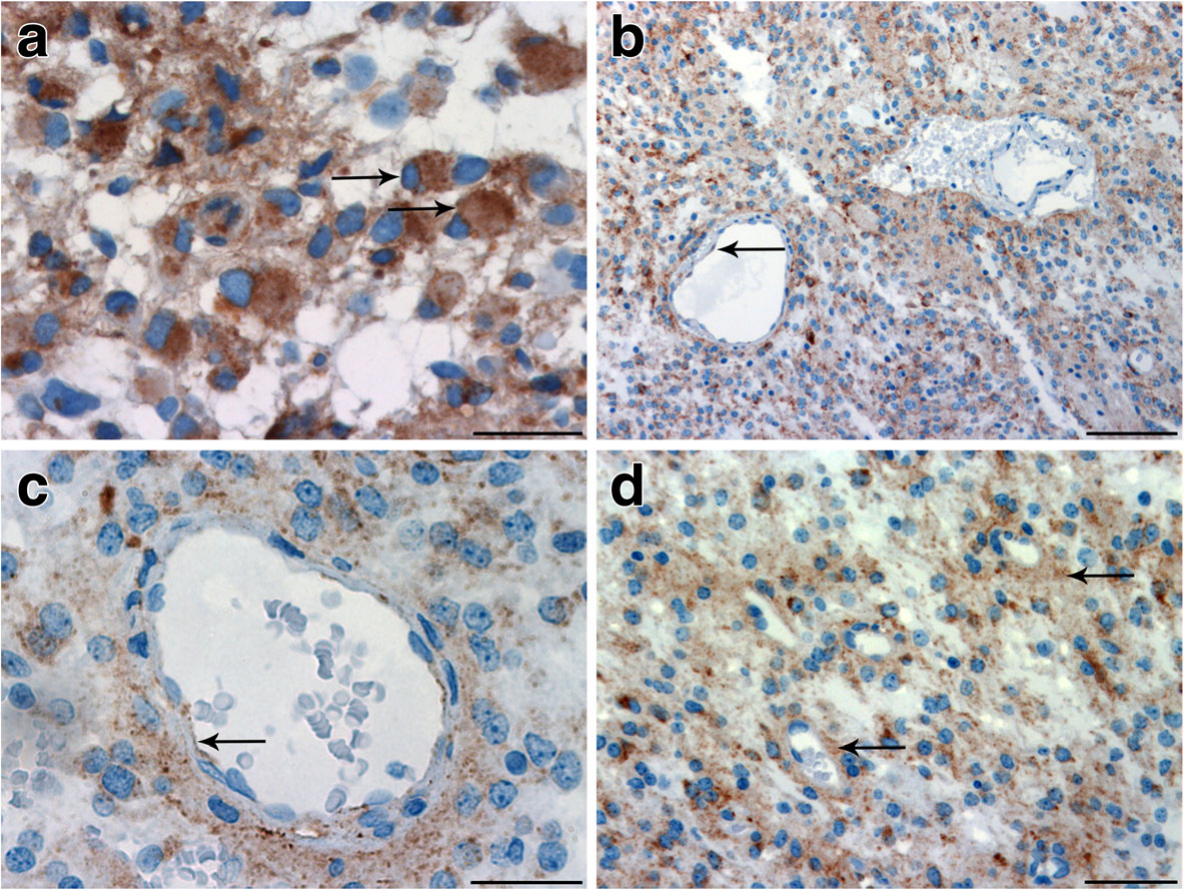
Immunohistochemical expression of CD63 in diffuse astrocytomas. **a** Gemistocytic tumors showed distinct CD63 expression (arrows), **b** whereas a more diffuse CD63 staining was seen in tumors with a fibrillary pattern. **b**, **c** A faint CD63 expression was detected close to the lumen of the some tumor blood vessels (arrows). **d** In some of the fibrillary tumors, an increased diffuse perivascular staining was observed (arrows). Scale bar: 30 μm (**a**, **c**), 100 μm (**b**), and 50 μm (**d**)

In diffuse astrocytomas with a gemistocytic pattern, tumor cells with distinct CD63 expression were observed (Fig. 1a), whereas a more diffuse CD63 staining was seen in tumors with a predominantly fibrillary pattern (Fig. 1b). Tumor blood vessels also expressed CD63, although not always adjacent to CD63+ tumor cells (Fig. 1b, c). In some of the blood vessels, CD63 was faintly expressed and mainly detected close to the lumen of the tumor blood vessels corresponding to the supposed localization of the apical endothelial membrane (Fig. 1c). An increased diffuse perivascular CD63 staining was observed in some of the fibrillary tumors (Fig. 1d); however, this did not appear to be related to CD63 labeling of the blood vessels.

### CD63 expression in anaplastic astrocytomas and glioblastomas

**Fig. 2:**
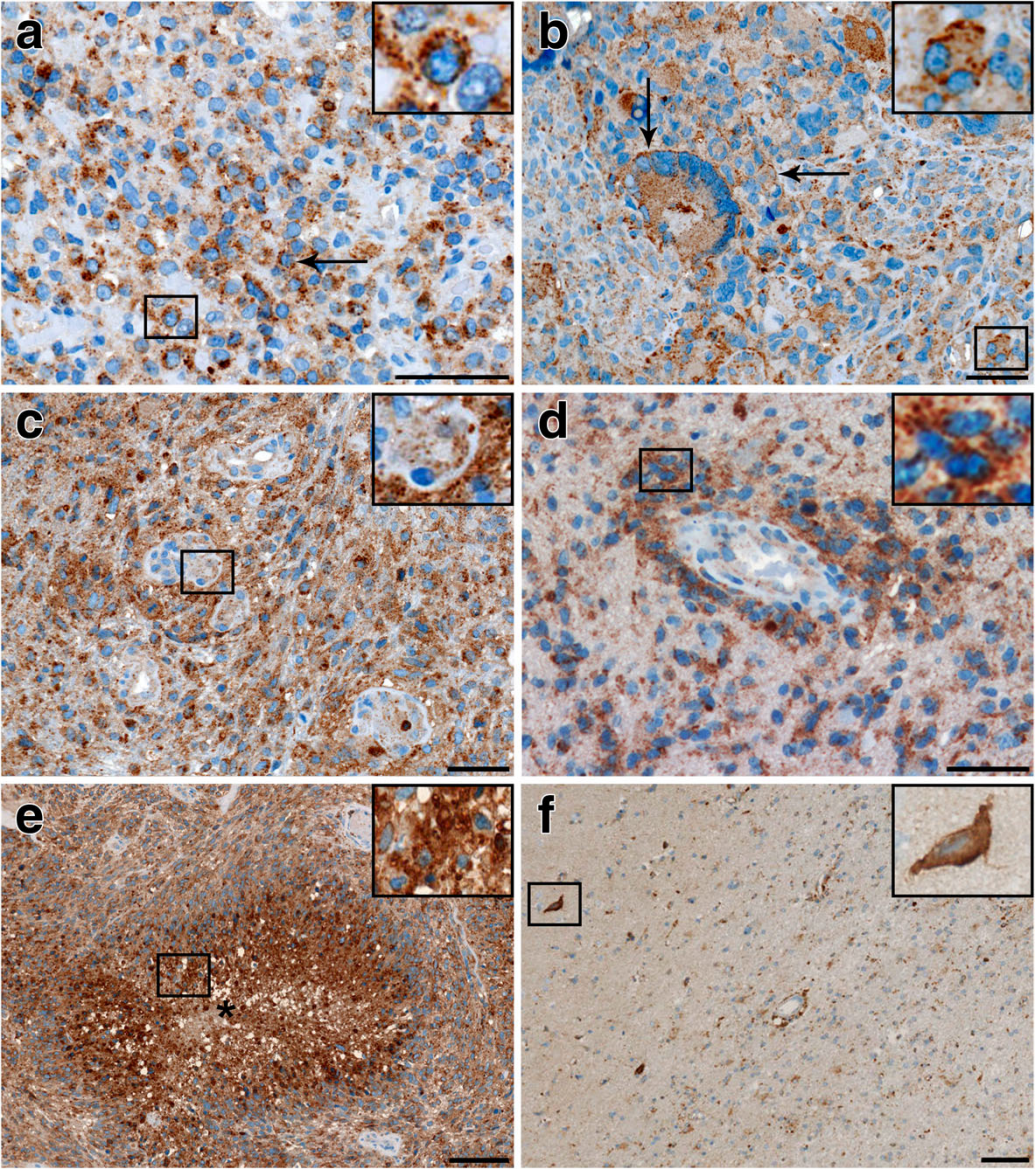
Immunohistochemical expression of CD63 in glioblastoma **a** A distinct cytoplasmic CD63 staining was seen in all tumors (arrow). **b** A distinct plasma membrane labeling was seen in some biopsies (arrows). **c**, **d** In general blood vessels showed a weak CD63 expression, but a more intense expression was found in perivascular tumor cells (d). **e** Pseudopalisade formations showed an intense CD63 expression. **f** A few CD63 tumor cells with faint CD63 expression as well as CD63+ pyramidal shaped neurons (arrow) were seen in the tumor periphery. Scale bar: 50 μm (**a**-**d**) and 100 μm (**e**-**f**)

In anaplastic astrocytomas and glioblastomas, the expression patterns of CD63 appeared similar, however, perinecrotic areas and proliferative blood vessels only expressed CD63 in glioblastomas. In all tumors, tumor cells showed a distinct punctate cytoplasmic CD63 expression (Fig. 2a). CD63 was detected in both fusiform and stellate shaped tumor cells as well as in multinucleated giant cells. In some areas, tumor cells also had a distinct labeling of the plasma membrane (Fig. 2b); while a more diffuse staining was found in other biopsies (Fig. 2c). CD63+ blood vessels were often located in relation to CD63+ tumor cells (Fig. 2c). Mostly, blood vessels stained weakly for CD63 (Fig. 2c-d). Similar to the diffuse astrocytomas, CD63 appeared to be located in the apical endothelial membrane as well as in the cytoplasm of endothelial cells. Also in some perivascular areas, tumor cells showed an intense CD63 expression (Fig. 2d). An intense CD63 expression was observed in pseudopalisade formations (Fig. 2e), while larger necrotic areas with cellular debris had a weaker and more diffuse CD63 expression (not shown). In some of the tumors, peripheral tumor areas were present, and these zones contained few diffusely invading and weakly CD63+ putative tumor cells as well as CD63+ pyramidal shaped neurons (Fig. 2f). Moreover, CD63+ blood vessels were also observed in the tumor periphery.

### CD63 expression levels in astrocytomas

**Fig. 3:**
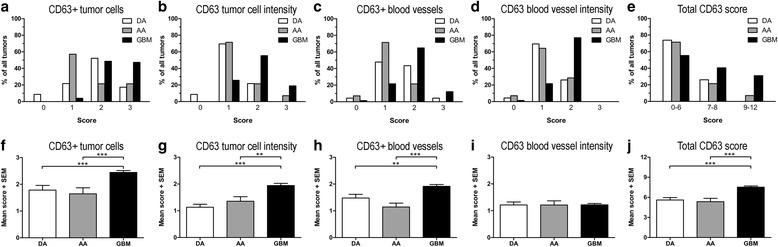
Expression levels of CD63 in astrocytomas Based on the CD63 expression, the astrocytomas received a score between 0 and 3 in four categories. Distributions are shown as percentage of the total number of tumors for the respective astrocytoma subtype for: **a** CD63+ tumor cells, **b** tumor cell intensity, **c** CD63+ blood vessels, **d** CD63 blood vessel staining intensity, and **e** total CD63 score. Comparison of the mean scores among the different WHO grades for: **f** CD63+ tumor cells, **g** tumor cell intensity, **h** CD63+ blood vessels, **i** CD63 blood vessel intensity, and **j** the total CD63 score. Data are shown as mean scores + standard error of the mean (SEM). **: *p* < 0.01; ***: *p* < 0.001. Abbreviations: AA anaplastic astrocytoma; DA diffuse astrocytoma; GBM glioblastoma

For the percentage of CD63+ tumor cells (Fig. 3a), tumor cell intensity (Fig. 3b), the percentage of CD63+ blood vessels (Fig. 3c), and CD63 blood vessel intensity (Fig. 3d), the highest scores 2 and 3 were most frequently given to glioblastomas, whereas the lowest score 1 was most often given to diffuse and anaplastic astrocytomas (Fig. 3a-d). However, very few tumors displayed clearly negative staining characteristics (Fig. 3a-d). In diffuse astrocytomas, CD63 was expressed by the tumor cells and the tumor blood vessels in 91% and 92% of the tumors, respectively. CD63+ tumor cells were present in all anaplastic astrocytoma and glioblastoma samples, while CD63+ blood vessels were detected in 93% of the anaplastic astrocytomas and 99% of the glioblastomas. Looking at the total CD63 score, 74% and 71% of the diffuse and anaplastic astrocytomas were in the lowest CD63 expression group, while this was only the case for 55% of the glioblastomas (Fig. 3e).

The amount of CD63+ tumor cells was similar in diffuse and anaplastic astrocytomas, while the amount was significantly higher in glioblastomas (*p* < 0.001) (Fig. 3f). CD63 tumor cell intensity also increased significantly with tumor grade (*p* < 0.01 and *p* < 0.001, respectively) (Fig. 3g). The level of CD63+ blood vessels tended to be lowest in anaplastic astrocytomas followed by diffuse astrocytomas, while glioblastomas had the highest score (*p* < 0.001 and *p* < 0.01, respectively) (Fig. 3h). The CD63 blood vessel intensity was similar for all grades (*p* = 1.00) (Fig. 3i). Comparing the total CD63 score, glioblastomas had significantly higher score compared to diffuse and anaplastic astrocytomas (*p* < 0.001) (Fig. 3j). When investigating the correlation between CD63 expression and IDH status, the level of CD63 tended to be higher in IDH wildtype tumors, but this was not significant (Additional file 1: Figure S1).

### The prognostic potential of CD63 in astrocytomas

**Fig. 4:**
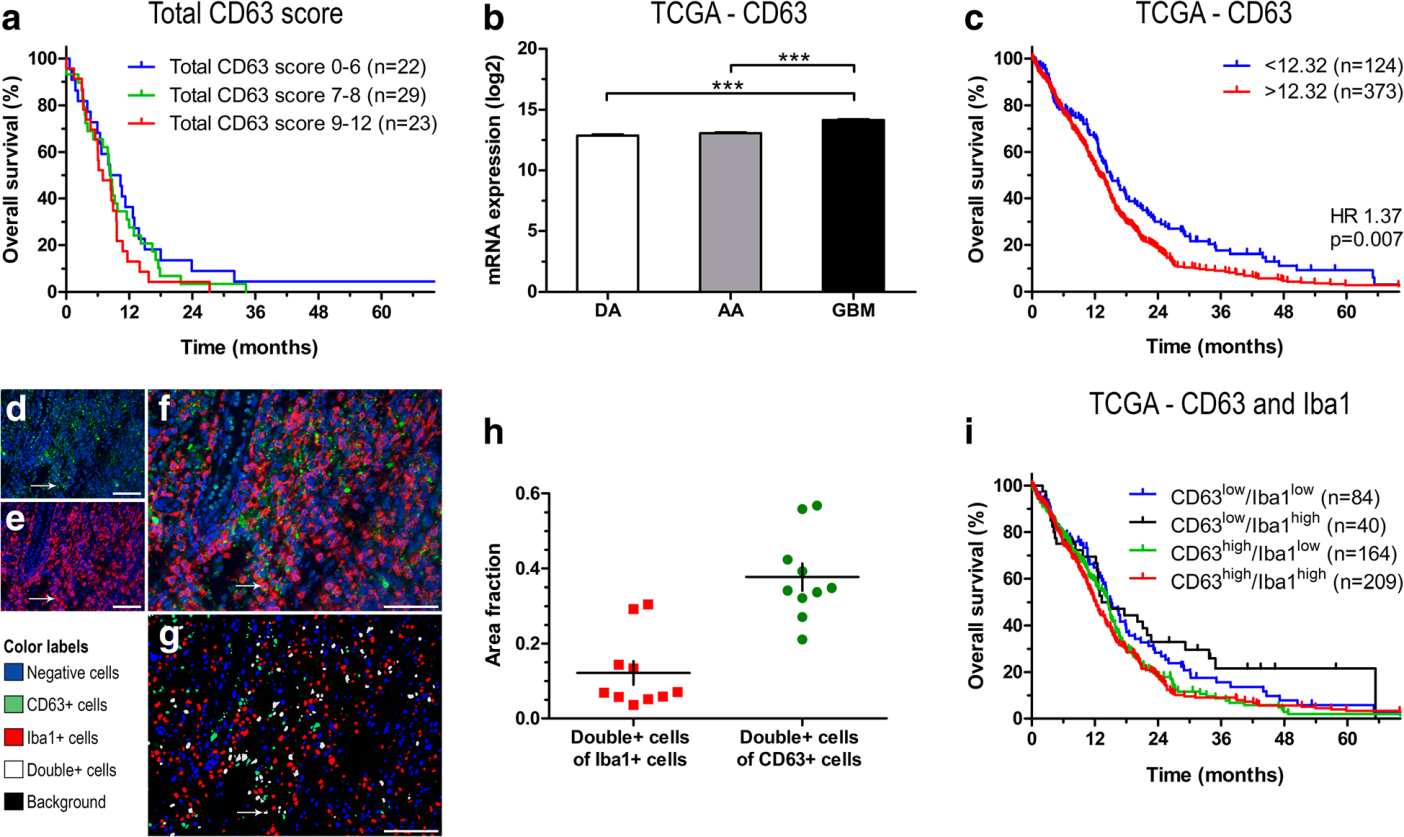
Association between CD63, Iba1, and prognosis in glioblastomas. **a** Kaplan-Meier curve for glioblastoma patients stratified into three groups based on total CD63 score. **b** Association between WHO malignancy grade and CD63 mRNA expression levels in the TCGA. **c** Using the TCGA dataset, low CD63 levels were significantly associated with improved survival in glioblastoma patients when dichotomized at the 25th percentile. **d-f** Double-immunofluorescence staining with CD63 (green) and Iba1 (red) using DAPI as nuclear staining (blue). **g** Software-based classification was performed using a threshold-based algorithm. Cells were assigned color labels based on their expression of CD63 and Iba1. Cells co-expressing CD63 and Iba1 are illustrated as white-labeled nuclei, cells only expressing Iba1 are depicted as red-labeled nuclei, cells only expressing CD63 are depicted as green-labeled nuclei, while negative cells are depicted as blue-labeled nuclei. **h** The areas of double-positive nuclei were calculated as fractions of the total Iba1+ nuclear area and the total CD63+ nuclear area. **i** Kaplan-Meier curve for glioblastoma patients included in the TCGA dataset stratified into four groups based on the CD63 and Iba1 mRNA expression levels. Scale bar: 100 μm. ***: *p* < 0.001. Abbreviations: AA anaplastic astrocytoma; DA diffuse astrocytoma; GBM glioblastoma

When investigating the association between overall survival and CD63+ tumor cell score, no significant correlation was found for patients with diffuse astrocytoma (*p* = 0.85), anaplastic astrocytomas (*p* = 0.31), or glioblastoma (*p* = 0.59). Similar results were found when examining the correlation between overall survival and tumor cell staining intensity (diffuse astrocytoma: *p* = 0.25; anaplastic astrocytoma: *p* = 0.24; glioblastoma: *p* = 0.88), CD63+ tumor blood vessel score (diffuse astrocytoma: *p* = 0.93; anaplastic astrocytoma: *p* = 0.74; glioblastoma: *p* = 0.27), as well as tumor blood vessel staining intensity (diffuse astrocytoma: *p* = 0.96; anaplastic astrocytoma: p = 0.74; glioblastoma: *p* = 0.73). Furthermore, no significant association between the total CD63 score and overall survival was detected in patients with diffuse (*p* = 0.11) or anaplastic (*p* = 0.19) astrocytoma. In glioblastoma, patients with the lowest CD63 expression (score 0–6; *n* = 22) tended to have a significantly longer overall survival than the patients with the highest CD63 expression (score 9–12; *n* = 23) (HR 1.61; 95% CI 0.88–2.96; *p* = 0.12), and similar tendency was found when adjusting for age and sex (HR 1.72; 95% CI 0.91–3.25; *p* = 0.095). No significant difference was found between the patients with low expression and the patients with medium expression (score 7–8; *n* = 29) in the univariate (HR 1.22; 95% CI 0.70–2.16; *p* = 0.48) or multivariate analysis (HR 1.20; 95% CI 0.68–2.14; *p* = 0.53) (Fig. 4a).

To further examine the impact of CD63 in astrocytomas, we used TCGA datasets. We found a grade-dependent increase in CD63 expression with glioblastomas having significantly higher levels compared to diffuse and anaplastic astrocytomas (*p* < 0.001) (Fig. 4b); also IDH wildtype tumors had significantly higher CD63 levels compared IDH mutated tumors (Additional file 1: Figure S1). At the median mRNA expression level, high CD63 was not significantly associated with shorter overall survival in patients with glioblastoma (HR 1.15; 95% CI 0.95–1.39; *p* = 0.15) (data not shown). However, dichotomized at the 25th percentile (i.e. the 25% patients with the lowest levels vs the 75% patients with the highest levels), patients with low CD63 mRNA levels had significantly better prognosis than patients with high levels (HR 1.37; CI 1.09–1.72; *p* = 0.007) (Fig. 4c). However, when adjusting for age and sex, this impact was not statistically significant (HR 1.20; 95% CI 0.95–1.51; *p* = 0.12).

To investigate the association between CD63 and immune cells, we performed automated quantitative double-immunofluorescence on ten glioblastomas (Fig. 4d-g). We found that the average fractions of double-positive cells were 0.12 of the total Iba1+ cells and 0.38 of the total CD63+ cells (Fig. 4h), and analysis showed a positive, yet insignificant, correlation between Iba1 and CD63 expression (Spearman’s ρ = 0.55; *p* = 0.098). In the TCGA dataset, a weak positive, but significant, correlation was found between Iba1 and CD63 mRNA levels (Spearman’s ρ = 0.19; *p* < 0.001). To examine the influence of Iba1 on the prognostic impact of CD63 in glioblastomas, patients included in the TCGA dataset were dichotomized based on the median mRNA levels of Iba1 and 25th percentile level of CD63 resulting in four groups: 1) CD63^low^/Iba1^low^, 2) CD63^low^/Iba1^high^, 3) CD63^high^/Iba1^low^, and 4) CD63^high^/Iba1^high^ (Fig. 4i). We found that patients with CD63^low^/Iba1^low^ mRNA levels tended to have longer overall survival compared to patients with CD63^high^/Iba1^high^ mRNA levels (HR 1.31; 95% CI 0.99–1.73; *p* = 0.059). However, no significant differences were found between the CD63^low^/Iba1^low^ and CD63^low^/Iba1^high^ groups (HR 0.83; 95% 0.53–1.28; *p* = 0.39) or between the CD63^high^/Iba1^low^ and CD63^high^/Iba1^high^ groups (HR 1.05; 95% 0.85–1.32; *p* = 0.64) suggesting that Iba1 levels do not impact the prognostic value of CD63 itself.

### The prognostic potential of CD63 and TIMP-1

**Fig. 5:**
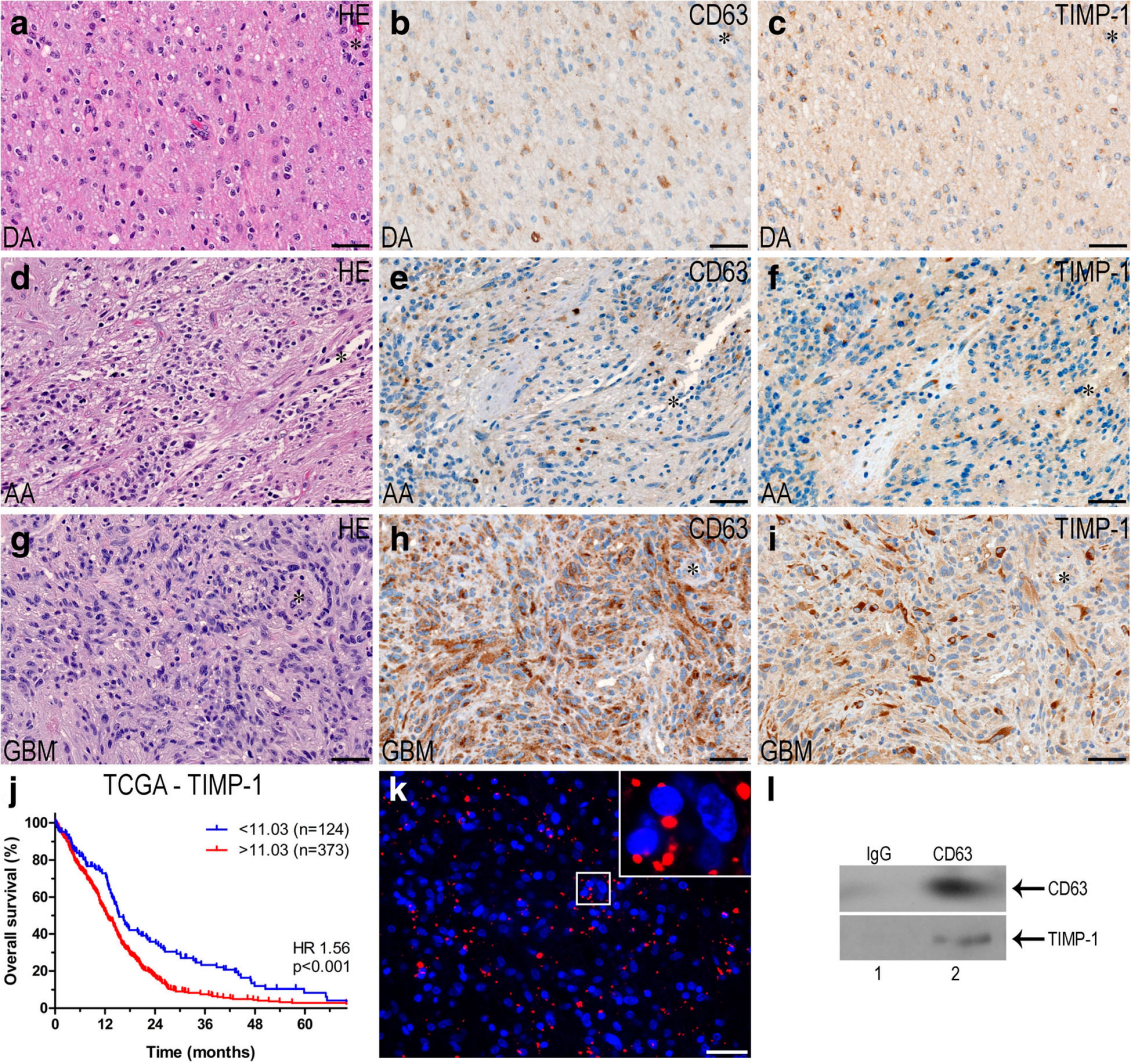
Association between TIMP-1 and CD63. **a-c** Representative haematoxylin-eosin, CD63, and TIMP-1 staining of a diffuse astrocytoma (DA). **d-f** Representative haematoxylin-eosin, CD63, and TIMP-1 staining of an anaplastic astrocytoma (AA). **g-i** Representative haematoxylin-eosin, CD63, and TIMP-1 staining of a glioblastoma (GBM). **j** Using the TCGA dataset, low TIMP-1 levels were significantly associated with improved survival in glioblastoma patients when dichotomized at the 25th percentile. **k** Proximity ligation assay performed on glioblastoma biopsies visualizing TIMP-1 and CD63 proteins in close molecular proximity. A red dot appears when proteins are located sufficiently close together. The nuclei were visualized with DAPI (blue color). **l** Immunoblotting using an anti-TIMP-1 antibody showing TIMP-1 and CD63 binding in U87MG cell line-derived spheroids. Cell lysates were immunoprecipitated with an anti-CD63 (lane 2) and the corresponding IgG isotype as control (lane 1). Scale bar 50 μm. *Asterisks* indicate blood vessels

Comparing the CD63 staining to the TIMP-1 staining, obtained in our previous study using the same cohort [14], the expression of CD63 and TIMP-1 appeared to show same grade-dependent increase (Fig. 5a-i). When performing Spearman’s correlation analysis, we found a weak, but significant, correlation between the total CD63 score and the total TIMP-1 score (Spearman’s ρ = 0.27; *p* = 0.024). Adding the TIMP-1 score as a variable in the regression model showed that CD63 expression did not impact the prognostic value of TIMP-1 (Table 3). Using the TCGA dataset, Spearman’s correlation analysis showed that CD63 mRNA levels were positively correlated with TIMP-1 mRNA levels (Spearman’s ρ = 0.60; *p <* 0.001). Investigating the association between survival and TIMP-1, we found that the 25% patients with the lowest TIMP-1 levels had significantly longer survival compare to the 75% with the highest levels (HR 1.56; 95% 1.23–1.97; *p* < 0.001) (Fig. 5j). This remained significant when adjusting for age and sex (HR 1.37; 95% CI 1.08–1.73; *p* = 0.008). Adjusting for both TIMP-1 and CD63 in the multivariate analysis showed that CD63 levels did not augment the prognostic value of TIMP-1 (HR 1.35; 95% CI 1.03–1.77; *p* = 0.027).

**Table 3:** Multivariate analysis of total CD63 score and total TIMP-1 score

		Baseline model		CD63	
	Patients (n)	HR (95% CI)	*P*-value	HR (95% CI)	*P*-value
Age (continuous)	74	1.01 (0.98–1.03)	0.70	1.00 (0.98–1.03)	0.71
Sex					
Male	47	1.00		1.00	
Female	27	0.82 (0.49–1.37)	0.44	0.76 (0.45–1.29)	0.31
TIMP-1 score					
0–5	12	1.00		1.00	
6–7	26	5.05 (2.14–11.9)	< 0.001	5.06 (2.13–12.0)	< 0.001
8–9	24	3.33 (1.43–7.71)	0.005	3.27 (1.38–7.76)	0.007
10–12	12	5.18 (1.96–13.6)	0.001	4.66 (1.74–12.5)	0.002
CD63 score					
0–6	22	–	–	1.00	
7–8	29	–	–	1.09 (0.58–2.06)	0.79
9–12	23	–	–	1.51 (0.78–2.91)	0.22

### Interaction between TIMP-1 and CD63

The proximity ligation assay showed TIMP-1 and CD63 proteins in close molecular proximity revealing these interacting molecules as red spots in the tumor cells of the glioblastoma biopsies (Fig. 5k). The same pattern was detected in the organotypic spheroids and in U87MG cell line-derived spheroids (not shown). To confirm this association, CD63 immunoprecipitation was performed with U87MG cell line-derived spheroids. Western blotting of isolated immunocomplexes from these cells, showed that anti-CD63 antibody co-immunoprecipitated CD63 together with TIMP-1, whereas the corresponding mouse isotype control IgG did not (Fig. 5l), indicating that TIMP-1 is associated with CD63 under the conditions of the assay. In summary, these assays suggest that TIMP-1 and CD63 are located closely together and interact in glioblastomas.

### TIMP-1 and CD63/CD133/Sox2 co-expression

**Fig. 6:**
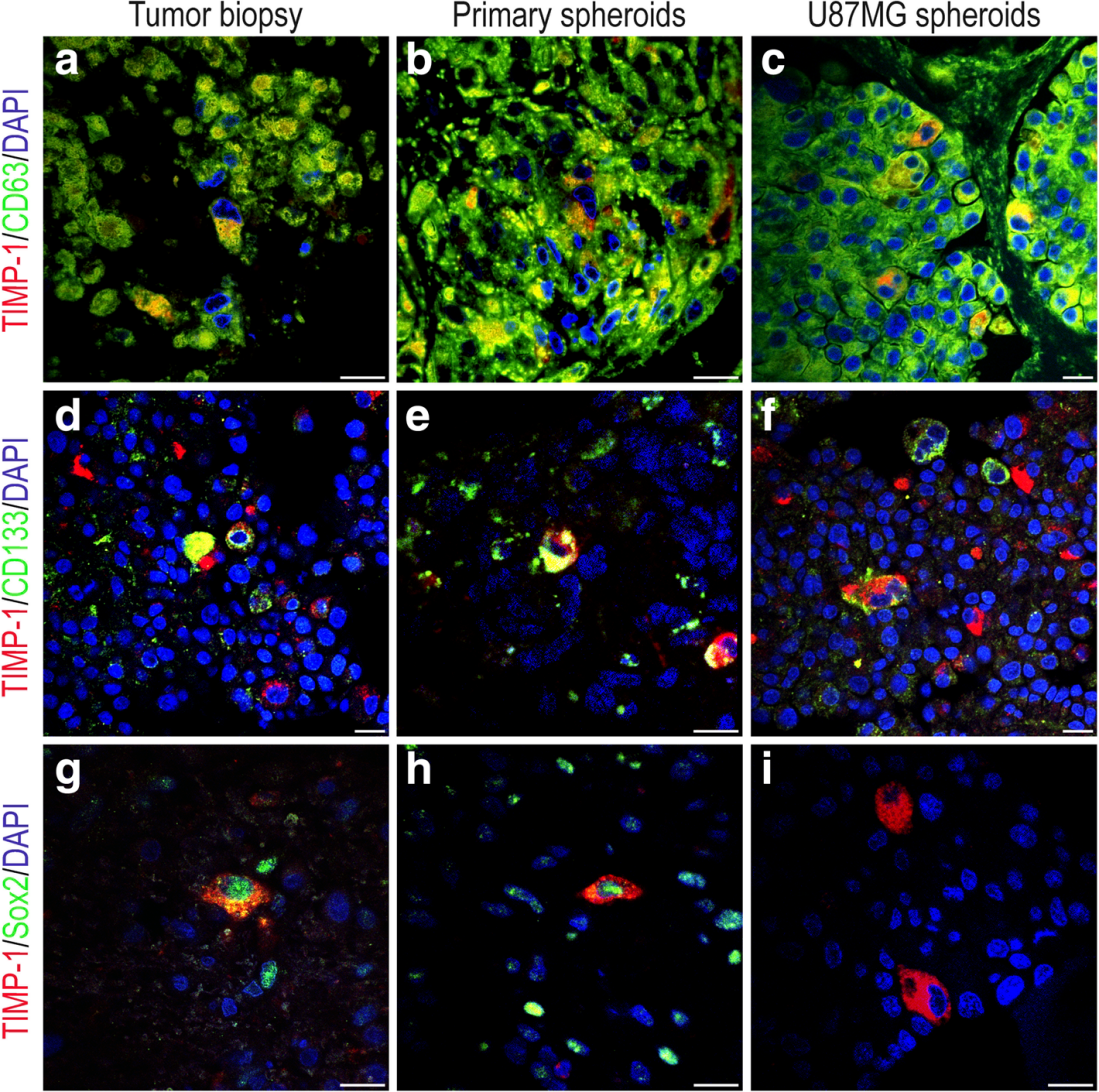
Double-immunofluorescence stainings of glioblastoma biopsies and spheroids. **a-c** Co-expression of TIMP-1 and CD63, **d-f** co-expression of TIMP-1 and the tumor stem cell-related marker CD133, and **g-i** co-expression of TIMP-1 and the tumor stem cell-related marker Sox2 in glioblastoma biopsies (**a**, **d**, **g**), cultured organotypic primary spheroids (**b**, **e**, **h**) and U87MG cell line-derived spheroids (**c**, **f**, **i**). TIMP-1/CD63 co-expression was detected in the biopsies (**a**) and spheroids (**b**, **c**). Only some tumor cells co-expressed TIMP-1 and CD133 in the biopsies (**d**) and spheroids (**e**, **f**). TIMP-1/Sox2 co-expression was detected in some tumor cells in the biopsies (**g**) and in the primary spheroids (**h**), whereas no Sox2 expression was detected in the U87MG cell line derived spheroids (**i**). Scale bar: 30 μm

TIMP-1/CD63 co-expression was detected in tumor biopsies (Fig. 6a) as well as in the organotypic (Fig. 6b) and U87MG cell line-derived spheroids (Fig. 6c). CD63 was widely expressed, while TIMP-1 expression was more infrequent, however, most TIMP-1+ cells were found to express CD63.

Some TIMP-1/CD133 co-expressing cells were detected in some of the tumor biopsies (Fig. 6d), in the corresponding organotypic spheroids (Fig. 6e) and in the U87MG cell line-derived spheroids (Fig. 6f), although the majority of the CD133+ cells did not co-express TIMP-1. Similar to this, TIMP-1/Sox2 co-expressing cells were detected in some of the tumor biopsies (Fig. 6g) and in the corresponding organotypic spheroids (Fig. 6h), although the majority of the Sox2+ cells did not co-express TIMP-1. No Sox2 expression was detected in the U87MG cell line (Fig. 6i). CD63 was distributed in virtually all tumor cells and spheroids and therefore, CD63 co-expression with stem cell markers was not specifically investigated.

### Differential expression analysis

**Fig. 7:**
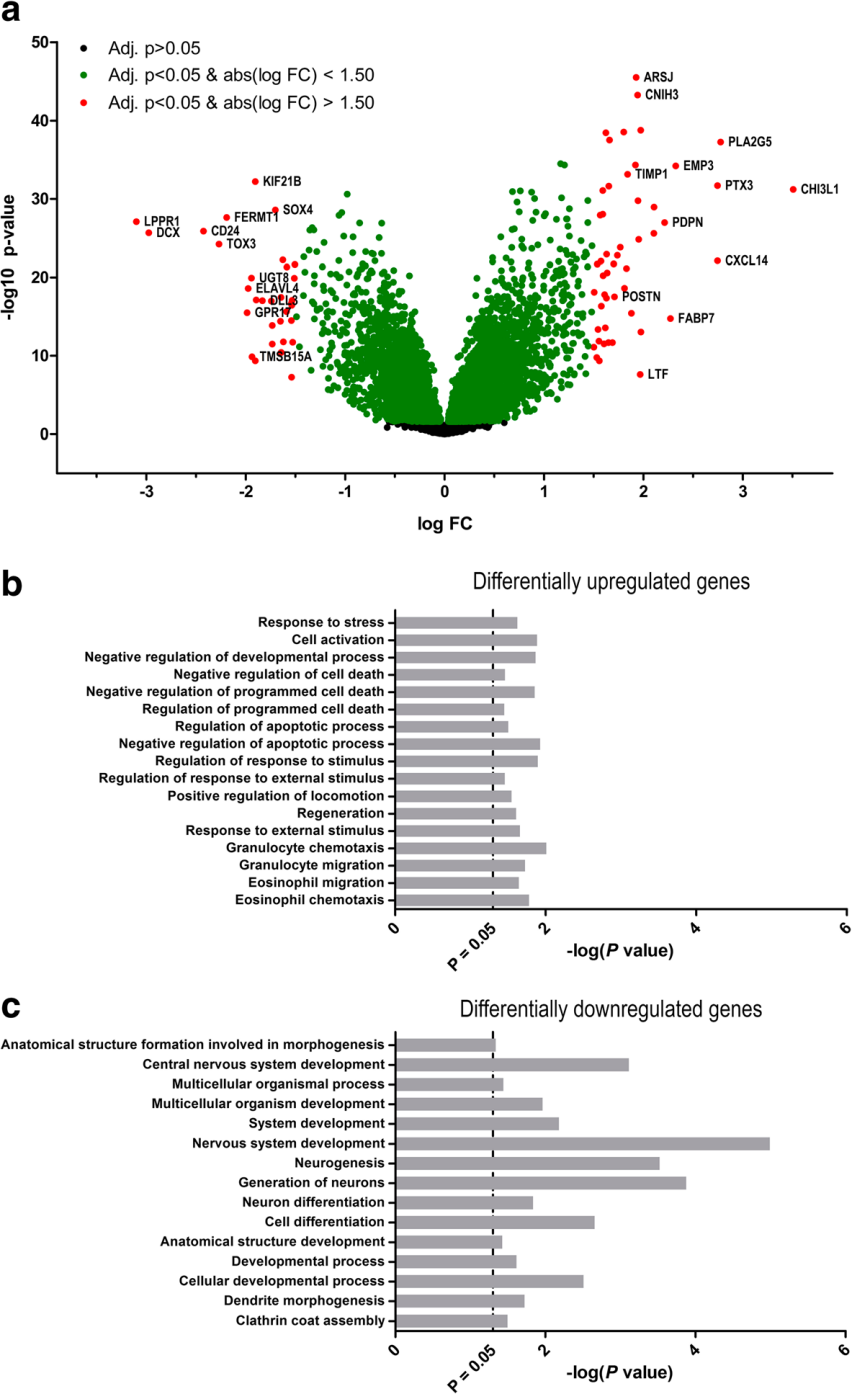
Differential expression analysis. **a** Volcano plot illustrating that 50 genes (red dots) were significantly upregulated above the 1.50-fold threshold, and that 32 genes (red dots) were significantly downregulated below the 1.50-fold threshold. **b-c** The up- and downregulated genes were hierarchically classified to biological processes using the Gene Ontology (GO) term enrichment analysis

To investigate the potential functional relevance of CD63 in glioblastoma, a volcano plot was generated using the TCGA dataset to visualize quantitative changes in mRNA expression levels of 12,700 genes between the groups of glioblastomas with the highest and lowest CD63 mRNA level (Fig. 7). A total of 50 genes were differentially upregulated (fold change > 1.50) including chitinase-3-like protein 1 (CHI3L1/YKL-40), TIMP-1, podoplanin (PDPN), fatty acid binding protein 7 (FAPB7), epithelial membrane protein 3 (EMP3), pentraxin 3 (PTX3), and periostin (POSTN), while 32 genes were downregulated (fold change > − 1.50); among these were delta-like 3 (DLL3), lipid phosphate phosphatase-related protein type 1 (LPPR1), and doublecortin (DCX) (Fig. 7a and Additional file 2: Table S1). GO term enrichment analysis was used to retrieve a biological profile of the differentially regulated genes; upregulated genes appeared to be involved in cell activation, regeneration, regulation of cell death, migration, and immune response (Fig, 7b), whereas downregulated genes appeared to be involved in cell differentiation and development, especially in the central nervous system (Fig. 7c).

## Discussion

In a previous study, we showed that TIMP-1 was a significant prognostic marker in glioblastomas, and in the present study we aimed to further investigate the role of TIMP-1 in glioblastomas by focusing on the potential TIMP-1 interacting protein CD63. We showed that the immunohistochemical expression of CD63 was highest in glioblastomas, but CD63 was not significantly associated with overall survival alone or in combination with TIMP-1, however, high levels tended to correlate with poorer prognosis. The TIMP-1 and CD63 proteins appeared to be located in close molecular proximity suggesting TIMP-1/CD63 interaction. This possible interaction has to our knowledge not been shown before in glioblastomas.

CD63 has previously been associated with cancer; however, the expression pattern differs dependent on the specific cancer type. In melanomas, high CD63 protein levels were associated with early stages of tumor progression and low levels with late disease stages [60], while CD63 mRNA expression levels were similar in pancreatic cancer and normal pancreatic tissue [61]. In lung adenocarcinomas, low CD63 levels relative to normal lung tissue were significantly associated with poorer survival for patients with grade I and II disease, but not for grade III [62]. In contrast, Kanao et al. showed that high CD63 mRNA expression was associated with a poor overall survival for stage I and II uterine cervical cancers [63].

In the present study, the immunohistochemical expression of CD63 protein was assessed in the same patient cohort that we previously used to examine TIMP-1 protein expression (14). We detected CD63+ tumor cells in the majority of the tumors and found a significantly higher CD63 expression in glioblastomas compared to diffuse and anaplastic astrocytomas. In contrast to the considerable variation observed in the TIMP-1 expression in glioblastomas [14], the variation in the CD63 expression was minor, and virtually all tumor cells were positive. The homogeneous CD63 expression in the biopsies partly explains the low correlation between TIMP-1 and CD63 observed in the present study. CD63 was also expressed in tumor blood vessels as well as in neurons in the tumor periphery. This may suggest that CD63 is important for tumor cell biology as well as for angiogenesis and neuronal survival.

Only few studies describe the presence of CD63 protein in gliomas using immunohistochemistry [37, 38]. Rorive et al. investigated the expression of CD63 on ten tissue microarrays consisting of 471 gliomas and found the CD63 expression to increase significantly with astrocytoma grade being significantly highest in glioblastomas and anaplastic astrocytomas compared to diffuse astrocytoma. In contrast, we found the highest CD63 expression in glioblastomas, but no difference was observed between anaplastic and diffuse astrocytomas. This discrepancy may be explained by a difference in the number of tumors with IDH mutation. Rorive et al. did not report IDH status, but our results suggest that astrocytomas with mutations in the IDH genes have lower CD63 levels compared to wildtype astrocytomas. Further, Rorive et al. found an association between patient survival and CD63 in glioblastomas, however, combining TIMP-4 and CD63 scores in the survival analysis showed that simultaneous expression of TIMP-4 and CD63 was a stronger negative prognostic factor compared to CD63 and TIMP-4 alone [37]. In the present study, high mRNA levels of CD63 were found to be associated with shorter overall survival using the TCGA dataset, but the association was not significant in the protein data. Also, CD63 expression did not affect the prognostic potential of TIMP-1 suggesting that TIMP-1 is a more valuable prognostic marker in glioblastomas compared to CD63 alone or in combination with CD63. The discrepancy between our results and the results by Rorive et al. could be explained by the number of patients included in the studies as Rorive et al. had 265 glioblastomas in their cohort, while our study only included 74 making it more difficult to detect a significant difference. The contradicting results could also be attributed to different staining protocols and scoring systems. In contrast to Rorive et al., we performed immunohistochemistry on whole tissue sections as expression of several markers may be under- or overestimated in tissue microarrays due to the heterogeneity of gliomas. Furthermore, TIMP-4 was only associated with prognosis when co-expressed with CD63 [37]; in contrast, we found that low TIMP-1 levels correlated with longer survival [14], and this finding was validated in the present study using TCGA data. Altogether, these results suggest that TIMP-1 is a stronger prognosticator in glioblastoma patients compared to CD63 and TIMP-4.

Recently, Kase et al. reported that a high number of CD63+ immune cells was associated with improved overall survival in glioblastoma patients [38]. In our study, we did not score CD63 expression in immune cells, but only in tumor cells and blood vessels. To address the possible expression of CD63 by immune cells, we performed double-immunofluorescence with CD63 and Iba1. We found that up to approx. 40% of the CD63+ cells co-expressed Iba1. To investigate the impact of Iba1 on the prognostic value of CD63, we used TCGA data and stratified patients based on the mRNA levels of CD63 and Iba1; Iba1 did not appear to influence CD63 substantially regarding patient survival. However, to fully elucidate the prognostic value of CD63+ immune cells and CD63+ tumor cells, additional prognostic studies should be performed e.g. by confocal double-immunofluorescence analysis. Overall, these conflicting results suggest that the specific cell type expressing CD63 may be important regarding prognosis.

In general, a weak CD63 expression was detected in a few putative invading tumor cells. This is in line with findings by Rorive et al. [37] and Wei et al. [64], as CD63 expression levels were found to be significantly higher in the tumor core compared to the tumor margin and periphery suggesting that CD63 mainly exerts its function in the bulk tumor. Whether CD63 plays a role in glioblastoma invasion needs further investigation. However, decreased levels of CD63 were found to enhance tumor motility and invasion in melanoma cell lines [29, 31]. In contrast, CD63 over-expression was reported to promote motility and frequency of metastases in a human uterine cervical cancer cell line [63], and CD63 knockdown was reported to increase invasion in vitro in esophageal carcinoma [65].

Using double-immunofluorescence, we showed that TIMP-1 and CD63 were co-expressed in some glioblastoma cells. Furthermore, the proximity ligation assay indicated that TIMP-1 and CD63 were located within a molecular distance suggestive of molecular interaction. This possible protein-protein interaction could have importance for the potential role of TIMP-1 in chemo-resistance [9–13]. Jung et al. identified CD63 as a possible TIMP-1 interacting protein, and over-expression of TIMP-1 in MCF10A cells resulted in inhibition of caspase-mediated apoptosis, while down-regulation of CD63 reversed the cellular protection mediated by TIMP-1 suggesting an important role for the TIMP-1/CD63 interaction in caspase-mediated apoptosis [28]. Whether this TIMP-1/CD63 interaction is present in glioblastomas is not known, however, our results suggest TIMP-1 and CD63 to be located in close molecular proximity, but additional investigations needs to be performed to elucidate possible biological roles of the TIMP-1/CD63 interaction in glioblastoma. However, Lee et al. found that TIMP-1 was a chemoattractant molecule enhancing the migration of neural stem cells towards glioma cells in vitro and showed that the migration was dependent on the expression of CD63 on the neural stem cells, overall suggesting an interaction of TIMP-1 and CD63 [41].

In the present study, we included spheroids to elucidate whether the co-expression of TIMP-1 and CD63, CD133 or Sox2 were preserved in cultured tissue and U87MG cell line-derived spheroids to determine if the spheroid models could be used in future studies investigating the role of TIMP-1 and CD63 in chemo-resistance. We found preserved expression of TIMP-1 and CD63, CD133 or Sox2 in the biopsies, and to some extent co-expression between the four proteins, suggesting that preclinical spheroid models can be used to determine the influence on chemo-resistance, also in relation to tumor stem-like cells which have been suggested to play an important role in resistance to anti-cancer treatment [2, 66–68]. Whether tumor stem-like cells in glioblastomas express CD63 has not been investigated directly in the present study as CD63 was expressed by virtually all tumor cells in the glioblastomas. Interestingly, we observed an increased density of CD63+ cells around blood vessels – similar to what has been reported for CD133+ tumor cells being localized in so-called perivascular niches [44].

Using the TCGA dataset, glioblastomas with the highest CD63 levels had a significant upregulation and downregulation of several genes compared to glioblastomas with the lowest levels. Among the differentially downregulated genes were DLL-3, DCX, and LPPR1. DLL-3 is associated with the proneural subtype of glioblastomas which is considered to be the least malignant and most treatment-sensitive of the molecular glioblastoma subtypes [69, 70]. LPPR1 is a member of the plasticity-related gene family influencing neuronal morphology [71, 72], but its potential function in gliomas is so far unknown. DCX was reported to inhibit glioma invasion in vitro [73] and reduce tumor volume in vivo [74] possibly by inducing apoptosis in tumor stem-like cells [75], overall indicating that DCX is a tumor suppressor. GO enrichment analysis of downregulated genes showed categories such as nervous system development and neurogenesis among the most enriched terms, thus consistent with the characteristics of the proneural glioblastoma subtype. Among the differentially upregulated genes were TIMP-1 as well as CHI3L1, PDPN, FAPB7, EMP3, PTX3, and POSTN. High levels of CHI3L1 and EMP3 [76] as well as FAPB7 [77, 78] and POSTN [79, 80] were reported as negative prognostic biomarkers in glioma. PTX3 [81], FAPB7 [77, 78], POSTN [79], and PDPN [82] are associated with increased glioma invasion, and both PDPN and CHI3L1 reportedly contribute to radio-resistance in glioblastoma [70, 83]. The upregulated genes were found to be especially involved in regulation of cell survival and cellular movement. CD63 is commonly used as a molecular marker of exosomes [32, 33, 35]; exosomes can provide an efficient and specific transfer of molecular signals between cells also in cancer, and components of both POSTN [84] and PDPN [85] are carried by exosomes along with other factors that facilitate angiogenesis, immune suppression, and oncogenesis [86]. In summary, these data indicate that CD63 is differentially over-expressed with molecules related to tumor aggressiveness and resistance and may thus contribute to a pro-tumorigenic microenvironment. The presence of CD63 in exosomes and exosomes in general could be used in the clinical setting as exosomes are carried in blood and shed into biological fluids, where it may have a potential as a diagnostic, predictive, or therapeutic tool [86].

## Conclusions

In conclusion, we found an increased expression of CD63 in glioblastomas as well as a weak correlation between CD63 and TIMP-1. However, the CD63 expression was not significantly correlated with prognosis alone or in combination with TIMP-1 at a protein level, but high levels tended to correlate with shorter overall survival. Interestingly, TIMP-1 and CD63 appeared to be located in close molecular proximity suggesting TIMP-1/CD63 interaction. The expression and co-localization was preserved in spheroids suggesting that spheroid models can be used in future studies investigating the possible biological role of the TIMP-1/CD63 interaction in chemo-resistance.

## Additional files

Additional file 1: **Figure S1**. Association between CD63 expression and isocitrate dehydrogenase (IDH) status. a Total CD63 score tended to be higher IDH wildtype tumors compared IDH mutated tumors. b CD63 mRNA expression levels were significantly higher in IDH wildtype tumors compared to mutated tumors. Abbreviations: AA anaplastic astrocytoma; DA diffuse astrocytoma. (TIFF 781 kb)

Additional file 2: **Table S1**. Differential expression analysis. List of differentially up- and downregulated genes in the group of glioblastomas with the highest CD63 mRNA levels compared to the group of glioblastomas with the lowest CD63 mRNA levels. (PDF 63 kb)

## Abbreviations

AIF: Allograft inflammatory factor 1
CHI3L1/YKL-40: Chitinase-3-like protein 1
CSA: Catalyzed Signal Amplification
DAB: Diaminobenzidine
DAPI: Diamidino-2-phenylindole
DCX: Doublecortin
DLL3: Delta-like 3
EMP3: Epithelial membrane protein 3
FAPB7: Fatty acid binding protein 7
GO: Gene ontology
HIER: Heat-induced epitope retrieval3
HSPC: Hematopoietic stem and progenitor cell
Iba1: Ionized calcium-binding adapter-molecule 1
IDH: Isocitrate dehydrogenase
LAMP-3: Lysosomal associated membrane protein 3
LPPR1: Lipid phosphate phosphatase-related protein type 1
PDPN: Podoplanin
POSTN: Periostin
PTX3: Pentraxin 3
Sox2: Sex determining region Y-box 2
TCGA: The Cancer Genome Atlas
TIMP: Tissue inhibitor of metalloproteinase
TSA: Tyramide Amplification Signal
WHO: World Health Organization
