# Supplementary figures and images for: Co-expression of TIMP-1 and its cell surface binding partner CD63 in glioblastomas
Source: BMC Cancer. 2018 Mar 9;18:270. doi: 10.1186/s12885-018-4179-y (PMC5845145; doi:10.1186/s12885-018-4179-y)

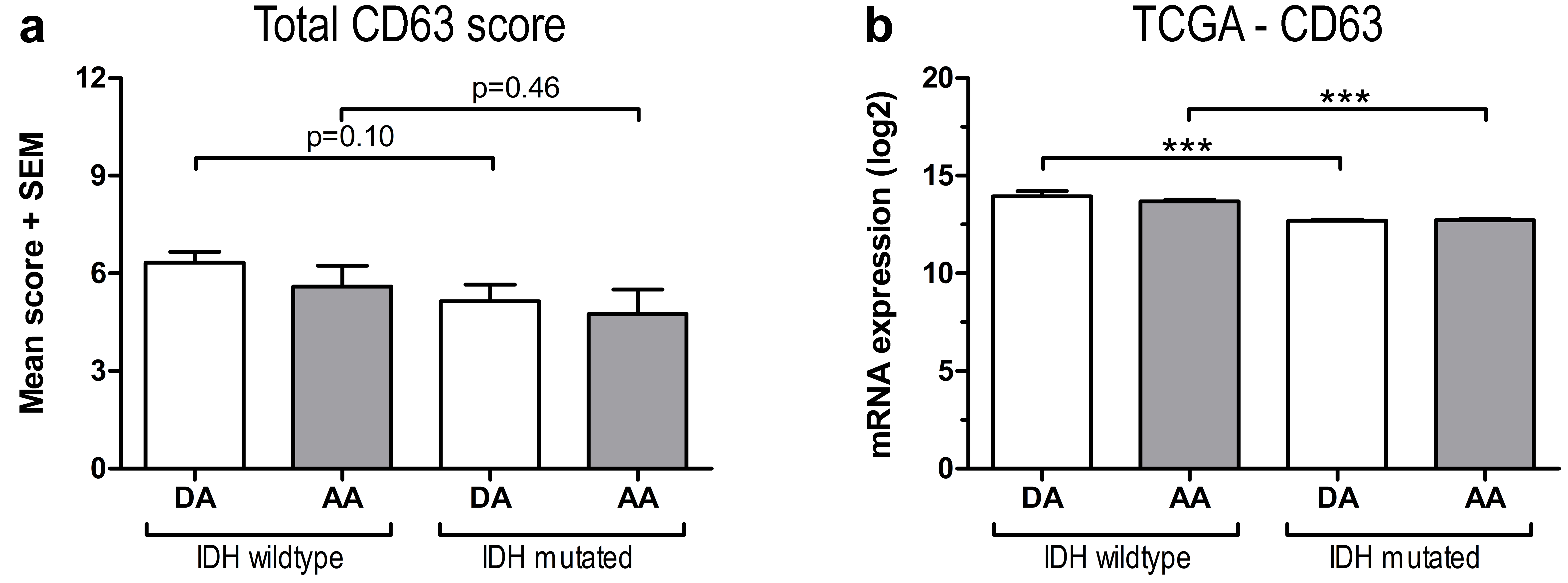

Supplement: Supplementary file 1 — Figure S1. Association between CD63 expression and isocitrate dehydrogenase (IDH) status. a Total CD63 score tended to be higher IDH wildtype tumors compared IDH mutated tumors. b CD63 mRNA expression levels were significantly higher in IDH wildtype tumors compared to mutated tumors. Abbreviations: AA anaplastic astrocytoma; DA diffuse astrocytoma. (TIFF 781 kb) [file 12885_2018_4179_MOESM1_ESM.tif]
