# Supplementary material for: Co-expression of TIMP-1 and its cell surface binding partner CD63 in glioblastomas
Source: BMC Cancer. 2018 Mar 9;18:270. doi: 10.1186/s12885-018-4179-y (PMC5845145; doi:10.1186/s12885-018-4179-y)
Supplement: Supplementary file 2 — Table S1. Differential expression analysis. List of differentially up- and downregulated genes in the group of glioblastomas with the highest CD63 mRNA levels compared to the group of glioblastomas with the lowest CD63 mRNA levels. (PDF 63 kb) [file 12885_2018_4179_MOESM2_ESM.pdf]

Table S1 List of 82 selected genes differentially expressed at the mRNA levels using the Cancer Genome Atlas (TCGA).

	log FC	Average expression	t	p-value	Adj. p-value
**Upregulated genes**					
CHI3L1	3.506922	10.53849	13.49852	6.10295E-32	4.55962E-29
PLA2G5	2.776492	6.475089	15.22195	5.27482E-38	8.37444E-35
CXCL14	2.744629	8.394921	10.82745	7.45708E-23	9.66453E-21
PTX3	2.7443	7.158813	13.63727	1.9998E-32	1.69329E-29
EMP3	2.324359	9.276723	14.36002	5.82214E-35	6.16225E-32
FABP7	2.270666	10.29682	8.464807	1.78696E-15	5.64582E-14
PDPN	2.212204	7.784825	12.27702	1.01757E-27	2.87205E-25
DIRAS3	2.106888	6.694933	12.85126	1.08046E-29	4.90103E-27
SRPX	2.104007	9.881435	11.87213	2.42772E-26	5.40956E-24
NNMT	1.97402	8.737411	7.864813	9.4515E-14	2.08861E-12
ACSS3	1.971636	6.411783	15.64735	1.64471E-39	5.22238E-36
LTF	1.967538	7.7558	5.74803	2.48316E-08	1.8829E-07
PIPOX	1.952258	7.66557	11.64092	1.46529E-25	2.90791E-23
CHRNA9	1.944729	5.615517	13.0853	1.67217E-30	8.16857E-28
CNIH3	1.940685	6.698849	16.91855	5.15477E-44	2.18236E-40
ARSJ	1.926178	5.752303	17.54484	3.14321E-46	1.9961E-42
LGALS3	1.919074	11.43747	14.38852	4.62132E-35	5.39012E-32
MOXD1	1.87921	7.122092	8.699329	3.62664E-16	1.29388E-14
TIMP1	1.840714	11.34029	14.05284	7.00008E-34	6.83907E-31
TNFAIP6	1.828614	7.424897	10.527	7.14996E-22	7.76167E-20
C21orf62	1.808542	6.379375	9.741257	2.33347E-19	1.56812E-17
ITGA7	1.80238	7.122253	15.58157	2.81257E-39	7.14449E-36
CA12	1.767784	7.37724	11.34693	1.41806E-24	2.50151E-22
KLHL4	1.737814	5.910478	11.03423	1.55225E-23	2.19057E-21
POSTN	1.708508	5.87961	9.384318	3.02742E-18	1.67179E-16
ANXA1	1.700277	10.26053	10.70289	1.90915E-22	2.26618E-20
CCL2	1.687438	8.854627	7.380556	2.04814E-12	3.43638E-11
HRH1	1.657925	6.946369	15.29494	2.91032E-38	5.28057E-35
EFEMP2	1.651725	8.075478	13.61924	2.31201E-32	1.83531E-29
MT1M	1.646948	8.826897	7.381991	2.02991E-12	3.4103E-11
DPYD	1.63535	8.327758	10.35296	2.61858E-21	2.48198E-19
C1R	1.629348	8.865473	11.08132	1.08417E-23	1.60117E-21
SERPINE1	1.627917	7.27665	9.336166	4.26308E-18	2.25606E-16
TNFRSF12A	1.62157	7.863064	15.55697	3.43738E-39	7.27635E-36
IGFBP2	1.61596	9.970375	8.054417	2.74627E-14	6.86621E-13
ELOVL2	1.608268	6.391563	9.476678	1.56648E-18	9.16859E-17
FCGBP	1.604532	9.521396	7.316951	3.0403E-12	4.95421E-11
LYPD1	1.594336	7.824418	10.24329	5.90689E-21	5.17402E-19
CCDC109B	1.590033	7.663051	13.45763	8.47578E-32	5.9806E-29
UPP1	1.589994	7.277932	12.58905	8.66238E-29	3.14345E-26
EFEMP1	1.575361	9.455032	8.996473	4.65248E-17	2.0099E-15

CLEC5A	1.572177	6.054744	10.81295	8.32138E-23	1.0569E-20
SPRY2	1.564268	9.383242	12.55494	1.1348E-28	4.00363E-26
CHI3L2	1.556502	7.750645	6.479946	4.46473E-10	4.76525E-09
SCG2	1.551559	8.769062	7.443392	1.38345E-12	2.39716E-11
HOPX	1.542805	11.25642	7.986759	4.27695E-14	1.02687E-12
FZD7	1.536535	7.338102	10.69889	1.9675E-22	2.31382E-20
MT3	1.529819	10.22169	6.652083	1.65366E-10	1.89036E-09
TMEM158	1.503772	9.511583	9.57446	7.77216E-19	4.76881E-17
CHL1	1.501739	8.207345	7.163432	7.81852E-12	1.17518E-10
**Downregulated genes**					
EPHB1	-1.5071	5.527674	-10.6785	2.29471E-22	2.67386E-20
EYA1	-1.51131	5.421593	-10.1436	1.23339E-20	9.91478E-19
KCND2	-1.52908	6.268968	-7.39769	1.8407E-12	3.11717E-11
DNM3	-1.53479	7.001034	-9.24256	8.2723E-18	4.13648E-16
DLX5	-1.53736	4.756159	-9.04119	3.40529E-17	1.48628E-15
AGXT2L1	-1.53999	6.73763	-5.59899	5.39418E-08	3.78935E-07
CA10	-1.54116	5.548342	-8.38416	3.07539E-15	9.25606E-14
RAB33A	-1.54131	6.351293	-9.12157	1.93941E-17	9.02288E-16
PAK3	-1.58681	6.307902	-10.5799	4.8091E-22	5.35792E-20
HMP19	-1.59019	5.403607	-8.76737	2.27342E-16	8.59365E-15
SCN3A	-1.62287	8.060168	-7.41223	1.68106E-12	2.86977E-11
BASP1	-1.62806	9.425308	-10.8609	5.78966E-23	7.65985E-21
SOX11	-1.64432	7.376329	-6.90681	3.68376E-11	4.8992E-10
DAAM2	-1.64559	6.899471	-9.36588	3.45164E-18	1.88152E-16
INA	-1.65203	5.924149	-8.34506	3.99727E-15	1.17792E-13
SOX4	-1.70343	7.261486	-12.7446	2.52173E-29	1.06762E-26
SH3GL2	-1.73391	6.38475	-8.15966	1.3728E-14	3.63596E-13
COL11A1	-1.73456	7.172921	-7.3075	3.22351E-12	5.22218E-11
FGF13	-1.74241	6.341501	-9.216	9.97842E-18	4.89328E-16
DLL3	-1.83234	6.403831	-9.23012	9.03229E-18	4.46378E-16
SNAP91	-1.8951	5.872425	-9.26094	7.2645E-18	3.64689E-16
KIF21B	-1.90414	5.841155	-13.7848	6.09322E-33	5.52786E-30
STMN2	-1.90421	7.106939	-6.48399	4.36269E-10	4.66811E-09
TMSB15A	-1.93779	7.953094	-6.685	1.36485E-10	1.58455E-09
UGT8	-1.94376	5.992744	-10.1481	1.19308E-20	9.77632E-19
ELAVL4	-1.97574	7.33395	-9.72752	2.57752E-19	1.71398E-17
GPR17	-1.9863	5.411438	-8.71951	3.1582E-16	1.13632E-14
FERMT1	-2.19254	6.356849	-12.4623	2.36033E-28	7.68682E-26
TOX3	-2.27034	6.860342	-11.4623	5.83303E-25	1.08949E-22
CD24	-2.42626	7.292749	-11.9554	1.26765E-26	2.92735E-24
DCX	-2.97604	6.873156	-11.8917	2.08478E-26	4.72836E-24
LPPR1	-3.10165	7.512313	-12.3092	7.89661E-28	2.27943E-25
